# Discovery of Fluorotelomer Sulfones in the Blubber of Greenland Killer Whales (*Orcinus orca*)

**DOI:** 10.1021/acs.estlett.5c00516

**Published:** 2025-08-13

**Authors:** Mélanie Z. Lauria, Xiaodi Shi, Faiz Haque, Merle Plassmann, Anna Roos, Malene Simon, Jonathan P. Benskin, Karl J. Jobst

**Affiliations:** † Department of Environmental Science, 7675 Stockholm University, Svante Arrhenius Väg 8, 106 91 Stockholm, Sweden; ‡ Department of Chemistry, 7675 Stockholm University, Svante Arrhenius Väg 16, 106 91, Stockholm, Sweden; § Department of Environmental Research and Monitoring, Swedish Museum of Natural History, 104 05 Stockholm, Sweden; ∥ Greenland Climate Research Centre, 114296 Greenland Institute of Natural Resources, 3900 Nuuk, Greenland; ⊥ Department of Chemistry, 428104 Memorial University of Newfoundland, 45 Arctic Ave., St. John’s, Canada A1C 5S7

**Keywords:** Combustion ion chromatography, gas chromatography ion mobility mass spectrometry, marine mammals, *Orcinus orca*, cetaceans, nontarget screening, PFAS

## Abstract

Most known per- and polyfluoroalkyl substances (PFAS) bioaccumulate by binding to proteins or partitioning to phospholipids, leading to their prevalence in liver and blood. However, the recent discovery of high concentrations of unidentified extractable organofluorine (EOF) in the blubber of a killer whale (*Orcinus orca*) from Greenland suggests that some fluorinated substances preferentially bioaccumulate in storage lipids. To further investigate this, the present work examined blubber from 4 killer whales (3 from Greenland, 1 from Sweden) via gas chromatography-atmospheric pressure chemical ionization-ion mobility mass spectrometry. Using collision cross sections, we prioritized features suspected to be highly fluorinated and then selected 5 for manual annotation. Custom synthesized standards confirmed 10:2 and 12:2 fluorotelomer methylsulfone, 10:2 and 12:2 fluorotelomer chloromethylsulfone, and 6:2 bisfluorotelomer sulfone in all blubber samples from Greenland at concentrations ranging from <0.4 to 72.5 ng/g, explaining 34–75% of blubber EOF, but none in the Swedish sample. None of these substances were observable in liver, suggesting preferential accumulation in storage lipids. To the best of our knowledge, this is the first report of neutral fluorotelomer sulfones in wildlife and the first identification of lipophilic, highly fluorinated PFAS.

## Graphical abstract

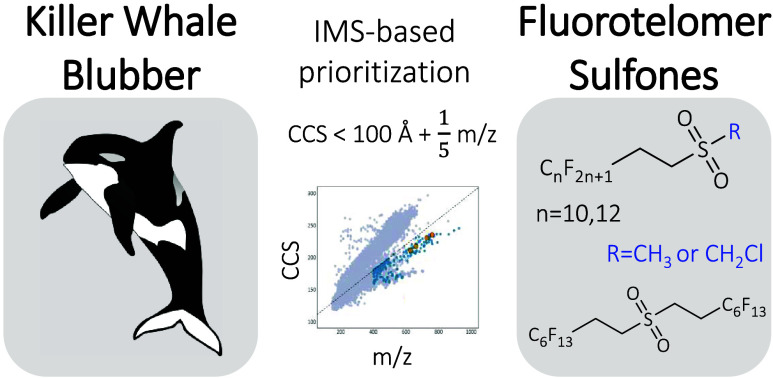

## Introduction

Chemicals containing fully fluorinated methyl (−CF_3_) or methylene (−CF_2_−) groups are classified as per- and polyfluoroalkyl substances (PFAS).^1^ These anthropogenic compounds have widespread industrial and consumer applications^2^ and to date, over 10 000 PFAS are known to exist on the global market, spanning both low-molecular weight water-soluble substances, to high molecular weight, hydrophobic polymers.^1^

Most PFAS research has focused on perfluoroalkyl acids (PFAAs) and their precursors. Among the most notorious PFAAs are perfluorooctanesulfonate (PFOS) and perfluorooctanoate (PFOA), highly water-soluble surfactants with low p*K*_a_s that accumulate in protein- and phospholipid-rich tissues (such as liver and blood) rather than in storage lipids like other persistent organic pollutants.^3–5^ While PFAA-precursors and alternatives (e.g., perfluorooctane sulfonamide and perfluorinated ether acids) have a greater propensity for fat partitioning compared to PFAAs, their concentrations in storage lipids remain lower than in liver or blood.^4,6,7^ For other PFAS, particularly neutral substances, tissue-specific accumulation remains either unexplored or is assumed to follow behavior similar to PFAAs. However, the impact of fluorination on lipophilicity is not always predictable. For instance, fluorination of an aromatic ring with either a single fluorine atom or a perfluoroalkyl group has been observed to increase lipophilicity compared to hydrogen at the same position, while fluorination of alkyl groups can lead to an increase or decrease in lipophilicity.^8,9^

Recently, the first empirical evidence of large quantities of unidentified extractable organic fluorine (EOF) in the blubber of a marine mammal was reported.^7^ In that work, a combination of combustion ion chromatography (CIC) and mass spectrometry-based target analyses were applied to eight different tissues of a killer whale (*Orcinus orca*) from East Greenland. While the distribution of known PFAS in tissues aligned with previous findings (with wet weight concentrations decreasing in the order: liver > blood > kidney ≈ lung ≈ ovary > muscle ≈ skin ≈ blubber), unknown EOF concentrations were highest in blubber. These results could not be explained by inorganic fluorine (which was removed during the extraction procedure) or targeted PFAS. Considering that blubber can account for up to 50% of the entire body mass of some species of cetaceans at certain life stages,^10,11^ we posit that overlooking chemicals in this compartment may significantly underestimate overall exposure to organofluorine substances.^12^

Efforts to characterize unidentified EOF have generally relied on suspect and nontarget screening using liquid chromatography-high resolution mass spectrometry (LC-HRMS) with electrospray ionization (ESI).^13–16^ This approach favors polar compounds which tend to be more easily ionizable, and is generally unsuitable for nonpolar/neutral substances, which do not ionize efficiently by ESI. To address this, several new methods have been developed based on gas chromatography-atmospheric pressure chemical ionization-high resolution mass spectrometry (GC-APCI-HRMS), which have proven effective at uncovering novel nonpolar PFAS in dust, water and sediment matrices.^17–20^ APCI is a softer ionization process compared to electron ionization, resulting in the detection of (quasi-)­molecular ions. Additionally, when coupled with ion mobility spectrometry (IMS), collision cross sections (CCSs) can be used as an additional prioritization strategy for fluorinated substances.^17^

In this work, we build on the initial discovery of unidentified EOF in Greenland killer whale blubber by characterizing EOF in an additional three individuals and identifying its origin using GC-APCI-IMS. To the best of our knowledge, this is the first study to identify lipophilic (i.e., occurring preferentially in storage lipids) highly fluorinated PFAS in wildlife.

## Methods and Materials

### Sample Collection

Blubber from three killer whales referred to herein as KW-16, KW-17 (previously characterized by Schultes et al.),^7^ and KW-20 were collected with local subsistence Inuit hunters in 2016, 2017, and 2020 in Greenland. Liver was also obtained from KW-17. Blubber from a fourth animal (KW-23), found dead at Hunnebostrand, Sweden, in 2023, was also sampled. Due to the rarity of strandings in Sweden, there is a sampling imbalance between the two locations. Additionally, as killer whales travel long distances, contaminant levels in a sampled individual may not reflect conditions at the location where it was found. Samples were kept frozen until extraction. Further details, including CITES permit numbers, are provided in Table S1 of the Supporting Information (SI).

### Sample Preparation

#### Extraction

Subsamples (2 g) of blubber (n = 3 for KW-17; n = 1 for all others) and liver (KW-17 only; n = 2) were thawed at room temperature and extracted with 4 mL of acetonitrile together with bead blending (4.8 mm stainless steel beads, 10 min at 1500 rpm, SPEX SamplePrep 1600 miniG). Subsequently, the samples were centrifuged (Centrifuge 5810, Eppendorf), and the supernatant was transferred to a new tube. The process was repeated, and the supernatants were combined and concentrated to 1 mL under nitrogen. A portion of extract (100 μL) was removed for EOF determination.

#### Lipid Removal

For characterization by GC-APCI-IMS (all 4 killer whales), extracts were subjected to a lipid removal procedure, described elsewhere and adapted here.^21^ Briefly, acetonitrile extracts were placed in a freezer (−24 °C) for 30 min to precipitate lipids. Thereafter the supernatants were filtered using a 0.45 μm nylon syringe filter and the filtrates were placed in a new tube. The procedure was repeated on precipitated lipids using 2 mL of acetonitrile, and the filtrates were combined.

#### Ion Exchange Cleanup

We hypothesized that lipophilic organofluorines would be neutral (i.e., lacking a net charge), and therefore sought to reduce the complexity of extracts by removing substances with ionizable functional groups (including but not limited to PFAAs). This was achieved using a series of cleanup steps based on ion exchange solid phase extraction (SPE). Strong cation exchange cartridges (Oasis MCX, 150 mg) were primed with 8 mL acetonitrile, filtrates (∼3 mL) were loaded and the cartridges were rinsed with an additional 8 mL of acetonitrile. The combined load and rinse were collected into a single tube and concentrated to ∼3 mL. This procedure was repeated with strong anion exchange cartridges (Oasis MAX, 150 mg). The final extracts were dried to ∼0.1 mL and transferred to microvials for GC-APCI-IMS analysis.

### Instrumental Analysis

#### Extractable Organofluorine Analysis

EOF was measured by CIC, using Mitsubishi combustion (HF-210) and gas absorption (GA-210) units coupled to a Thermo Scientific ion-chromatograph as described previously.^7,14^ Details are provided in the SI.

#### GC-APCI-IMS

Putative identification of fluorinated substances in KW-17 blubber extracts was carried out at Memorial University (Newfoundland, Canada), using an existing GC-APCI-IMS method for nontarget discovery of halogenated substances.^17^ Product ion spectra were obtained with a collision energy of 50 V for selected masses prioritized as likely to be highly fluorinated (see Data Analysis for details). These results were later replicated on all 4 killer whales and built upon at Stockholm University using a recently developed GC-APCI-IMS method with several modifications,^19^ details of which can be found in the SI.

### Quality Control

Method blanks for both CIC and GC-APCI-IMS analyses were determined by carrying out the same extraction procedure as for the samples in duplicates using empty tubes. Solvent blanks were always used in between samples in both CIC and GC-APCI-IMS to monitor carry-over. Analysis of Certified Reference Material (fluorine in clay, 568 ± 60 μg F/g, n = 3) and a solution of PFOS and PFOA (0.74 ng F/μL) were used to check for combustion efficiency throughout the CIC run, resulting in fluorine recoveries of 90% ± 9% and 105% ± 2%, respectively.

In addition, portions of extract from KW-17 were retained after lipid removal and again after ion exchange. Analysis of these extracts by CIC revealed that EOF concentrations remained stable with each successive step, indicating that the major fluorinated substances in blubber were not inadvertently removed during cleanup.

### Data Analysis

Lockmass correction, peak picking (minimum absolute ion intensity: 40), run alignment, calculation of CCSs, and pairing of precursor and product ions (minimum intensity: 1% of parent ion) were carried out using Progenesis QI (version 3, Waters corporation). Peaks with areas at least 10 times higher than the area in the procedural blanks were kept for screening. Features were selected for further investigation if their CCSs were between 150 and 250 Å^2^ and were lower than one-fifth of their *m*/*z* + 100 Å^2^, an approach previously demonstrated to be effective for prioritizing fluorinated and other halogenated substances.^17^ The resulting short-listed features were further prioritized based on a) exact mass >400 Da (which we posited could serve as a threshold for bioaccumulative PFAS, given that long chain PFAAs have masses exceeding 400 Da), and b) mass defects between −0.1 and +0.05 (characteristic of highly fluorinated substances).^22^ Finally, the most intense features were prioritized for manual inspection and annotation and if a structure was assigned, the presence of its homologues was manually checked by looking for masses increasing or decreasing in increments of 50 Da (CF_2_). Increasing CCS with retention time was used as additional evidence for the presence of a homologue. Custom synthesized standards for putatively identified compounds were purchased from Chiron AS (Trondheim, Norway) and used for confirmation and quantification. More information on these substances (e.g., chemical names, purity) can be found in Table S2.

### Quantification

Peak areas for quantification were obtained via Waters UNIFI software. Concentrations for the fluorotelomer sulfones identified in KW-17 were calculated by standard addition. Matrix effects were calculated as the ratio between the concentration calculated with external one-point calibration (500 ng/mL mixture of five fluorotelomer sulfones) and the concentration calculated by standard additions (Table S3). For the other killer whales, semiquantification was performed using the external one-point calibration and concentrations were adjusted by the matrix effect calculated in KW-17. PFAS concentrations (in ng/g) were converted to fluorine equivalent concentrations (i.e., ng F/g) and summed to compare with EOF measurements (see data handling in the SI). LOQs were estimated from a low concentration standard by estimating the concentration associated with a peak height of 500 (deemed the lowest signal that could be reasonably considered a peak). These concentrations were then adjusted by their respective matrix effect factor to obtain an LOQ for each substance (Table S3).

## Results and Discussion

### EOF Determination

EOF was measured in blubber of KW-16 (69 ng F/g, n = 1), KW-17 (162 ± 94 ng F/g, n = 6) and KW-20 (82 ng F/g, n = 1), but was below the LOQ for KW-23 (Figure 1, panel A and Table S3). The EOF concentration in KW-17 reported here represents the average of triplicate measurements in this study and previous measurements by Schultes et al. (2020).^7^ Measurements of EOF after cleanup (EnviCarb) in Schultes et al. and before any cleanup steps in the present work may explain the slight discrepancy in EOF concentrations between the two studies. The low level in KW-23 could be associated with the state of the animal, whose blubber appeared less dense in fats and had a higher relative proportion of connective tissue, suggesting that it may have died of starvation. It is unknown how this might influence contaminant levels and analytical results. Moreover, despite being only 18 years old, this individual exhibited unusually worn teeth, suggesting a diet rich in sharks (species with abrasive, sandpaper-like skin).^23,24^ This feeding behavior likely differs from that of the Greenlandic individuals which typically feed at a higher trophic level, consuming other highly contaminated marine mammals, and may help explain the absence of EOF in the Swedish sample, as dietary habits are known to play a more important role than location in driving pollutant exposure in killer whales.^25,26^

**Figure 1:**
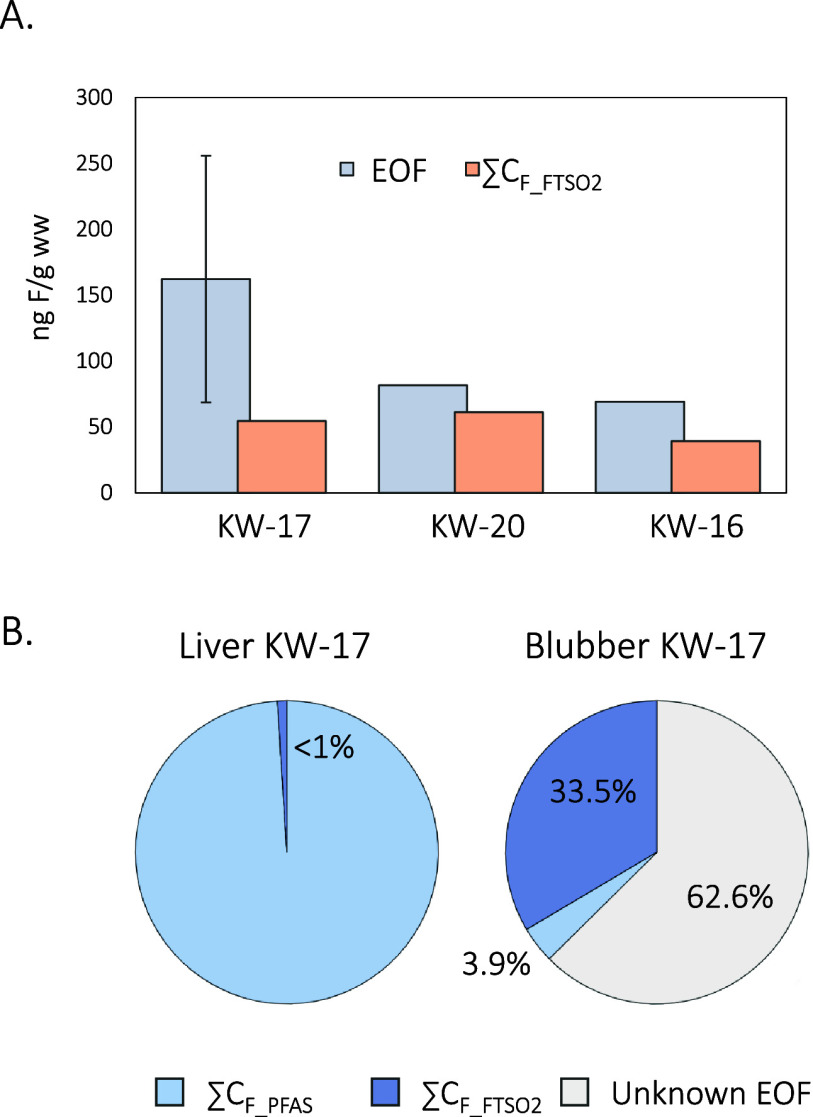
(A) EOF (gray) and ∑C_F_FTSO2_ (sum of concentrations of fluorotelomer sulfones in fluorine equivalents, orange) in ng F/g wet weight, measured in the blubber of Greenland killer whales. Data for KW-23 (from Sweden) are not shown because EOF and fluorotelomer sulfones were below LOQs. (B) Percentage of EOF in blubber and liver of KW-17 explained by ∑C_F_PFAS_ (in light blue, the sum of concentrations of PFAS measured by LC-HRMS by Schultes et al.,^7^ in F equivalents) and ∑C_F_FTSO2_ measured by GC-APCI-IMS in this study (blue), along with remaining unidentified EOF (gray). In the liver, ∑C_F_FTSO2_ concentrations were estimated using their LOQs, resulting in a value that is less than 1% of the EOF.

### HRMS Characterization

A total of 17043 features (>10× the abundance in method blanks) were observed in the KW-17 extract prior to any prioritization steps. Application of the CCS prioritization reduced the total number of features to 3910, which was further reduced to 344 features by selecting masses >400 Da which also displayed a mass defect between −0.1 and +0.05. Features were then ordered by intensity, and 5 were selected for structural elucidation (overview in Table 1, Figure 2, spectra in Figures S1–S4).

**Figure 2:**
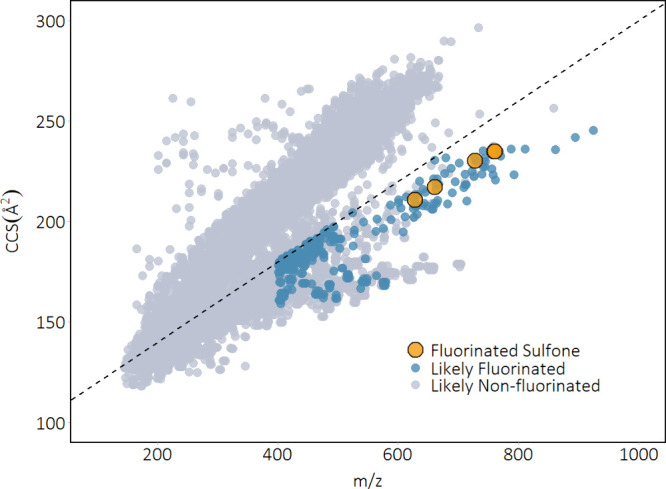
Plot of mass-to-charge ratios (*m*/*z*) versus collision cross section (CCS) of peaks detected in KW-17. Peaks with a CCS value under the threshold (100Å^2^ + 0.2 × *m*/*z*; denoted by the dashed line) and between 150 and 250 Å^2^ were prioritized as potential halogenated compounds. Further prioritization as possible fluorinated substances (in blue) utilized criteria of *m*/*z* > 400, and mass defect between −0.1 and +0.05. Identified features (the five fluorotelomer sulfones) are in orange (*m*/*z* 759 and 761 overlap).

**Table 1:**
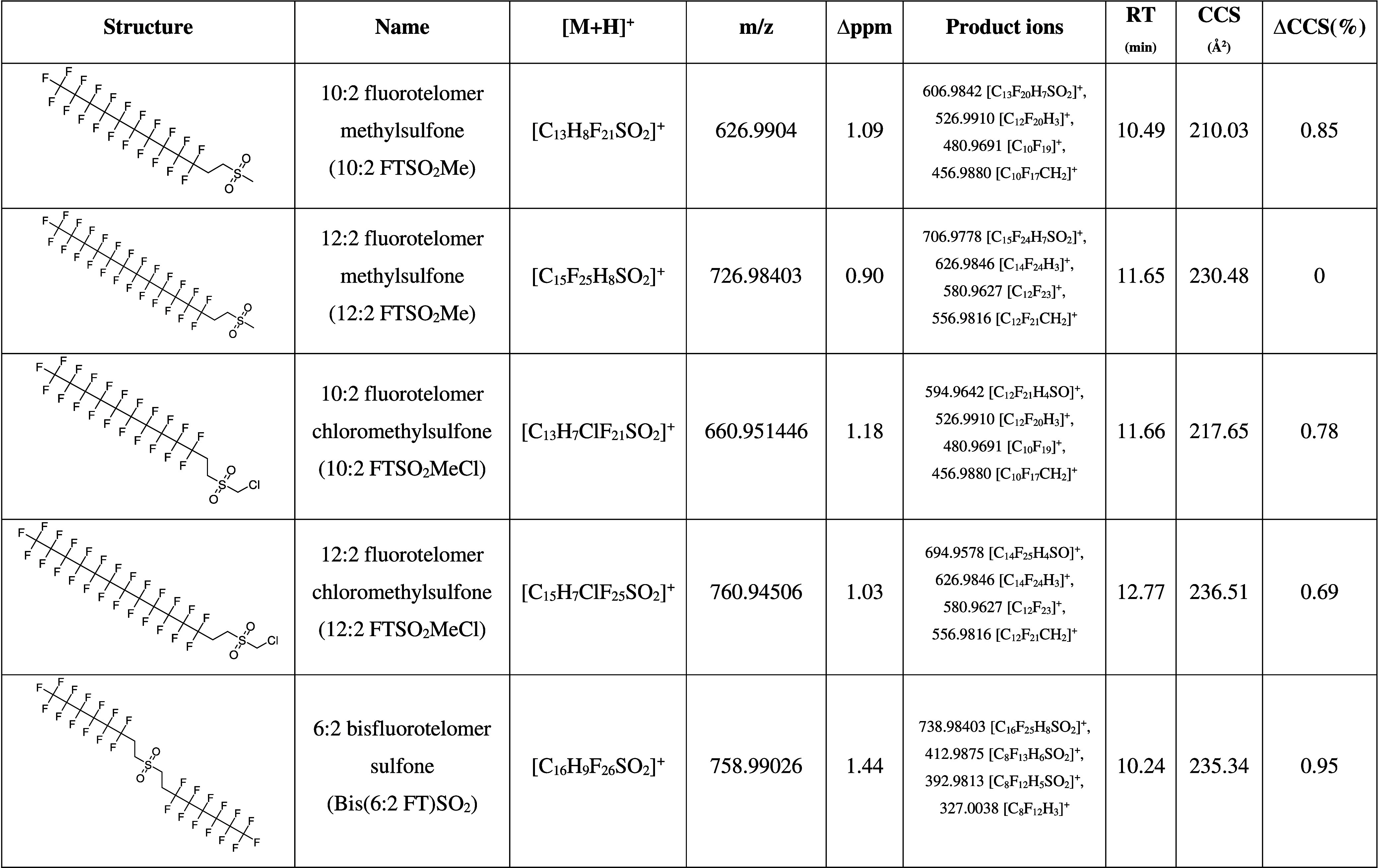
Structures, Names, and Acronyms of Fluorotelomer Sulfones, Formula, and Calculated *m/z* of [M+H]^+^, Average ppm Error of Observed *m/z* in Killer Whales, Major Product Ions Used for Structural Elucidation, Retention Time (RT), Average CCS Value in the Standard, and Average CCS Relative Deviation (%) in Killer Whales

The two most abundant peaks (*m*/*z* 627 and 727), were putatively identified as protonated ions of 10:2 fluorotelomer methylsulfone (10:2 FTSO_2_Me; [C_13_H_8_F_21_SO_2_]^+^) and 12:2 fluorotelomer methylsulfone (12:2 FTSO_2_Me; [C_15_H_8_F_25_SO_2_]^+^). Inspection of the product ion spectra for *m*/*z* 627 and 727 (Figure S1) revealed loss of HF, producing fragments [C_13_H_7_F_20_SO_2_]^+^ and [C_15_H_7_F_24_SO_2_]^+^, respectively, and subsequent loss of SO_2_CH_4_, forming fragments [C_12_F_20_H_3_]^+^ and [C_14_F_24_H_3_]^+^, respectively. From these, subsequent fragmentation follows either loss of C_2_H_2_ and another HF giving fragments [C_10_F_19_]^+^ and [C_12_F_23_]^+^, respectively, or loss of CHF_3_ resulting in fragments [C_10_F_17_CH_2_]^+^ and [C_12_F_21_CH_2_]^+^, respectively. Other smaller fluorinated fragments were also present in the product ion spectrum (Figure S1). Finally, further investigation of the chromatograms led to tentative identification of 2 additional homologues (6:2 and 8:2), based on increasing RT and CCS values with *m*/*z* (Figure S5 and Table S3), albeit at much lower abundances than the 10:2 and 12:2 species.

Following putative identification of the methyl sulfones, 10:2 fluorotelomer chloromethylsulfone (10:2 FTSO_2_MeCl) and 12:2 fluorotelomer chloromethylsulfone (12:2 FTSO_2_MeCl) were tentatively identified at lower intensities. These substances were noticed because 10:2 FTSO_2_MeCl appeared to partially coelute with 12:2 FTSO_2_Me. Fragmentation patterns followed loss of CH_3_OCl, producing [C_12_F_21_H_4_SO]^+^ and [C_14_F_25_H_4_SO]^+^, or loss of the sulfone chloromethyl head and HF, giving major fragments [C_12_F_20_H_3_]^+^ and [C_14_F_24_H_3_]^+^, respectively. From these, additional fragmentation follows the same pattern as for FTSO_2_Me (Figures S2 and S3).

The third most abundant peak after prioritization was *m*/*z* 759, which was putatively identified as 6:2 bisfluorotelomer sulfone (bis­(6:2 FT)­SO_2_). Fragments observed in the product ion spectrum (Figure S4) are due to loss of HF [C_16_F_25_H_8_SO_2_]^+^, loss of one of the fluorotelomer sulfone chains [C_8_F_13_H_6_SO_2_]^+^, and a subsequent loss of HF, giving fragment [C_8_F_12_H_5_SO_2_]^+^ which is itself followed by loss of S­(OH)_2_ resulting in [C_8_F_12_H_3_]^+^. Further investigation of the chromatograms led to tentative identification of 3 additional homologues (4:2, 5:2, 7:2), based on increasing RT and CCS values with *m*/*z* (Figure S5 and Table S4), albeit at much lower abundances than the 6:2 species.

### Confirmation, Quantification, and Organofluorine Mass Balance

Analysis of standards confirmed the identities of the five fluorotelomer sulfones reported in Table 1 in KW-17 at a sum concentration (∑C_FTSO2_) of 83.9 ng/g, made up of 23.6 ng/g 10:2 FTSO_2_Me, 55.2 ng/g 12:2 FTSO_2_Me, 1.1 ng/g 10:2 FTSO_2_MeCl, 0.4 ng/g 12:2 FTSO_2_MeCl and 3.6 ng/g bis­(6:2 FT)­SO_2_. None of these targets were observable in liver from the same animal. Subsequent analyses revealed similar sum concentrations to KW-17 for both KW-16 (60.3 ng/g) and KW-20 (94.0 ng/g), with 12:2 FTSO_2_Me displaying the highest concentrations, followed by 10:2 FTSO_2_Me, bis­(6:2 FT)­SO_2_ and 10:2 FTSO_2_MeCl. For 10:2 FTSO_2_MeCl, peaks were detectable but areas were below LOQ. KW-23 was the only animal where these targets were not observed. Detailed concentrations can be found in Table S3.

Conversion of ∑C_FTSO2_ to fluorine equivalents (∑C_F_FTSO2_) revealed concentrations of 39.2, 54.4, and 61.2 ng F/g, for KW16, 17, and 20, accounting for 57%, 34%, and 75% of the EOF in these animals, respectively. Schultes et al.^7^ previously measured polar PFAS (e.g., perfluoroalkyl sulfonates and carboxylates) by LC-HRMS in KW-17 blubber extracts, which accounted for only 6.3 ng F/g (4% of EOF). When combined with fluorotelomer sulfone concentrations, 37% of EOF was explained in KW-17 blubber (Figure 1, panel B), suggesting that additional fluorinated compounds still remain to be identified in blubber. In comparison, if we hypothesize that these compounds are present in the liver at their LOQs, they would account for <1% of the liver EOF, which is unsurprising considering that the fluorine mass balance was already closed by polar PFAS (Figure 1B).

### Implications

To the best of our knowledge, this is the first report of highly fluorinated nonpolar PFAS in marine mammal blubber. Due to the small sample size in the present study, the prevalence of this finding requires confirmation, ideally with optimized extraction procedures and targeted instrumental methods. Nevertheless, we have subsequently confirmed the n:2 FTSO_2_Me (n = 8, 10, 12, 14) in sediment samples from the Baltic Sea, the Arctic, a Norwegian lake contaminated by PFAS from paper production, and NIST reference material (1941b-Organics in Marine Sediment)^20^ at confidence levels 1 and 2.^27^ n:2 FTSO_2_Me homologues (n = 6, 8, 10) were also tentatively (i.e., without standards) reported in wastewater treatment plant influent, effluent and river water from Spain.^18^ Sources are unclear but several possibilities exist. One is that these substances are transformation products. Methyl sulfone metabolites of legacy chlorinated POPs, including PCBs, DDT, PCDDs, and PCDFs have been widely reported in humans and several marine mammal species.^28–31^ These metabolites are formed through Phase I/II metabolism to a glutathione conjugate, which is further processed via the mercapturic acid pathway to a cysteine thiol intermediate. Methylation and oxidation of the thiol yields the methyl sulfone.^32^ Sulfinyl and sulfone-containing PFAS (not including the substances reported here) have also been observed from biotransformation of thioether-containing aqueous film forming components (e.g., fluorotelomer thioether amido sulfonate).^33–35^ Alternatively, these substances could be production impurities or byproducts. Exposure may have occurred via long-range transport, or through feeding in source regions, the latter being relevant for more mobile killer whale populations.^26^

Regardless of the source, the occurrence of these chemicals in Greenland killer whale blubber suggests novel bioaccumulative behavior, as they were not observed above quantification limits in the liver, challenging the paradigm that all PFAS bioaccumulate through protein or phospholipid interactions. Moreover, estimates of whole-body burden suggest that the total mass of fluorotelomer sulfones in killer whales may be over twice that of conventional PFAS (i.e., 86 mg F for ∑C_F_FTSO2_ versus 38 mg F for ∑_24_PFAS), assuming a 4500 kg total mass (35% blubber/3.5% liver;^11,36–40^ see SI for calculations), highlighting the importance of these substances in overall PFAS exposure assessment.

Given that a considerable portion of EOF remains unexplained and more than 300 features prioritized as possible PFAS remain unidentified, further investigation is necessary. Nevertheless, this study marks a significant advancement in understanding the composition of EOF in lipid-rich tissues and highlights the importance of including nonpolar PFAS in fluorine mass balance studies and in environmental exposure considerations.

## Supplementary Material



## Notes

Details on chemicals, reagents, instrumental analysis, data handling and whole-body burden calculations; MS/MS figures, information on killer whales sampled, and standards of fluorotelomer sulfones, detailed measured concentrations of fluorotelomer sulfones and extractable organofluorine as well as information on additional homologues not confirmed with standards. (PDF)
