# Supplementary material for: Discovery of Fluorotelomer Sulfones in the Blubber of Greenland Killer Whales (Orcinus orca)
Source: Environ Sci Technol Lett. 2025 Aug 13;12(9):1218–24. doi: 10.1021/acs.estlett.5c00516 (PMC12424159; doi:10.1021/acs.estlett.5c00516)
Supplement: Supplementary file 1 [file ez5c00516_si_001.pdf]

Supporting Information

Discovery of Fluorotelomer Sulfones in the Blubber of Greenland Killer Whales (*Orcinus orca*)

Mélanie Z. Lauria,[1*] Xiaodi Shi, [1] Faiz Haque,[1, 2] Merle Plassmann,[1] Anna Roos,[3,4] Malene Simon,[4] Jonathan P. Benskin,[#1*] and Karl J. Jobst [#5*]

[1]Department of Environmental Science, Stockholm University, Svante Arrhenius Väg 8, 106 91 Stockholm, Sweden

[2]Department of Chemistry, Stockholm University, Svante Arrhenius Väg 16, 106 91, Stockholm, Sweden

[3] Department of Environmental Research and Monitoring, Swedish Museum of Natural History, 104 05 Stockholm, Sweden

[4] Greenland Climate Research Centre, Greenland Institute of Natural Resources, 3900 Nuuk, Greenland

[5]Department of Chemistry, Memorial University of Newfoundland, 45 Arctic Ave., St. John's, Canada A1C 5S7

[#]JPB and KJ contributed equally and share the last authorship.

*Corresponding authors:

Melanie.Lauria@aces.su.se

Jon.Benskin@aces.su.se

kjobst@mun.ca

Chemicals and reagents

Acetonitrile was obtained from Honeywell (France), acetone and toluene were purchased from Merck (Germany). Water was purified using a Millipore water purification system, resulting in a resistance of less than 18 MΩ/cm (Milli-Q water). The fluoride standard (1000 mg/L) was sourced from Thermo Scientific. EnviCarb (Supelclean™) and the certified reference material (CRM) fluorine in clay (BCR-461) was acquired from Sigma Aldrich. Stainless steel beads (4.8 mm) were purchased from Next Advance©. Argon and oxygen gases used for combustion ion chromatography (CIC) analysis were of purity grade 5.0. Fluorotelomer sulfones standards were custom made by Chiron AS (Trondheim, Norway) and prepared in a mixture (500 ng/mL) of acetone and toluene (25% and 75% respectively). More information on the standards can be found in Table S2.

Instrumental analysis

Extractable organofluorine analysis

For EOF determination by CIC, extracts (100 µl) were loaded into prebaked ceramic sample boats containing glass wool. The samples were combusted at 1100 °C with oxygen (400 mL/min), argon (200 mL/min), and an argon/water vapor mix (100 mL/min) within the combustion unit (HF-210, Mitsubishi) for five minutes. During the combustion process, combustion gases were absorbed in Milli-Q water using a gas absorber unit (GA-210, Mitsubishi), they were subsequently separated and analysed using an ion-chromatograph (Dionex, Thermo Scientific). Quantification was accomplished using calibration points at 0.05, 0.1, 0.25, 0.5, 1, 5 and 10 ppm of NaF solution, employing an unweighted linear calibration curve. Analysis of Certified Reference Material (fluorine in clay, 568 ± 60 µg F/g, n=3) and a solution of PFOS and PFOA (0.74 ng F/µl) were used to check for combustion efficiency throughout the run, resulting in recoveries of 90%±9% and 105%±2%, respectively. Mean fluoride concentrations from procedural blanks was subtracted from samples and the limit of quantification (LOQ; 4.7 ng F/g) was calculated using 3 times the standard deviation of replicate blank measurements, the average extract volume and average sample weight.

GC-APCI-IMS

Putative identification of fluorinated substances in KW-17 blubber extracts was carried out at Memorial University (Newfoundland, Canada), using an existing GC-APCI-IMS method for non-target discovery of halogenated substances[1] and product ion spectra were obtained with a collision energy of 50 V for selected masses. These results were later replicated and built upon at Stockholm University using a recently developed GC-APCI-IMS method with few modifications.[2] Briefly, extracts (1 µL) were injected onto an Agilent GC using pulse splitless mode with a programmed inlet temperature (i.e., initially 100 °C for 0.15 min, increased at 600 °C min-1 to 280 °C, hold for 1 min). Analytes were separated on a 30m DB-5MS Ultra Inert column (i.d., 0.25 mm; film thickness, 0.25 µm; Agilent Technologies) with helium carrier gas at a constant flow of 1.5 mL min-1. The GC oven temperature

program was as follows: hold at 70 °C for 1 min; increase at 10 °C min-1 to 310 °C, then hold for 15 min (total run time=40 min). The GC was coupled via an APCI source to a Waters Select Series Cyclic IMS operated in positive ionization mode and under wet conditions (an open vial containing water was placed in the source and left to equilibrate overnight). The transfer line and ion source were maintained at 290 °C and 150 °C, respectively. The corona discharge needle and cone voltage were set at 2 µA and 30 V, respectively. Nitrogen was used for the makeup-, auxiliary-, and cone gas at flow rates of 200 mL min-1, 150 L h-1, and 200 L h-1, respectively, under wet conditions. The MS was operated in the high-definition MSE mode with the mass range of 100-1200 amu. The collision energy was fixed at 6 eV at the low energy mode, and ramped between 15-50 eV at the high energy mode. The scan time was 0.3 s for each mode. The cyclic ion mobility cell was operated in the one pass mode with 5 pushes per bin at a traveling wave height of 15 V. Both drift gas and collision gas were nitrogen. Column bleeding (C9H27O5Si5+: m/z 355.0705) was measured every 2 min for internal mass calibration. CCS were calibrated using a mixture of 22 compounds supplied by Waters Corp. according to its standard procedure.

Analytical challenges in GC-APCI-MS analysis of fluorotelomer sulfones

Since the method used was not specifically optimized for fluorotelomer sulfones, a number of analytical challenges were encountered when analysing analytical standards for the first time. Initially, the analysis was carried out under dry conditions, producing both $M^{+\cdot}$ and $[M+H]^+$ ions. However, it quickly became apparent that the ratio between these two was unstable. For this reason, we chose to carry out analyses for quantification under wet conditions, favouring formation of $[M+H]^+$ ions. It is important to allow sufficient time for the source to equilibrate under such conditions, and that the ion ratio is monitored throughout long analytical sequences. A second problem was due to high concentrations of two of the fluorotelomer sulfones (10:2 and 12:2 $FTSO_2Me$) in samples, which led to suspected backflash-induced carryover. The carryover signal did not disappear from instrumental blank runs until the injector port and septa were thoroughly cleaned. This observation was surprising since the vapour volume of the extract injected was estimated to be only ~60% of the liner volume. It is therefore recommended to include solvent blanks in between samples to monitor for possible ghost peaks caused by backflash.

Data handling

Concentrations of compounds ($C_{\text{Fluorotelomer sulfones}}$ in ng/g) were converted to corresponding fluorine concentration ($C_{\text{F\_Fluorotelomer sulfones}}$ in ng F/g) and summed ($\sum C_{\text{F\_Fluorotelomer sulfones}}$) in order to compare with EOF measurements according to equation 1.

Eq 1. $C_{\text{F\_Fluorotelomer sulfones}} = n_F \times MW_F / MW_{\text{Fluorotelomer sulfones}} \times C_{\text{Fluorotelomer sulfones}}$

$C_{\text{F\_Fluorotelomer sulfones}}$ = The fluorine equivalent concentration for a fluorotelomer sulfone

n_F = The number of fluorine atoms in the fluorotelomer sulfone

MW_F = The atomic weight of fluorine

$MW_{Fluorotelomer\ sulfones}$ = The monoisotopic weight of the fluorotelomer sulfone.

$C_{Fluorotelomer\ sulfones}$ = The measured fluorotelomer sulfone concentration using GC-APCI-cIMS

Whole-body burden calculations

Depending on factors such as age, sex, and nutritional status, the liver of cetaceans can constitute approximately ~2% to 5% of the total body weight, while blubber can account for ~20-50%.[3–5] Whole body weights typically range from ~3000-4000 kg for adult females and ~5000-6000 kg for adult males.[6] Whole-body burden calculations assumed a mass of 4500 kg, consisting of 35% blubber and 3.5% liver. Sum fluorotelomer sulfone concentrations ($\sum C_{F\_Fluorotelomer\ sulfones}$; ng F/g) measured in blubber of KW-17 (i.e. 54.5 ng F/g) were then multiplied by the assumed total mass of blubber (1 575 000 g) followed by unit conversion to obtain a value of 86 mg F. Sum 24 PFAS concentrations ($\sum_{24}$PFAS; ng F/g) measured previously in liver of KW-17 (i.e. 240 ng F/g)[7] were multiplied by the assumed total mass of liver (157 500 g) followed by unit conversion to obtain a value of 38 mg F.

References

1. MacNeil A, Li X, Amiri R, et al. Gas Chromatography-(Cyclic) Ion Mobility Mass Spectrometry: A Novel Platform for the Discovery of Unknown Per-/Polyfluoroalkyl Substances. *Anal Chem*. 2022;94(31):11096-11103. doi:10.1021/acs.analchem.2c02325
2. Shi X, Sobek A, Benskin JP. Multidimensional-Constrained Suspect Screening of Hydrophobic Contaminants Using Gas Chromatography-Atmospheric Pressure Chemical Ionization-Ion Mobility-Mass Spectrometry. *Anal Chem*. 2025;97(10):5434-5438. doi:10.1021/acs.analchem.4c06234
3. Bories P, Rikardsen AH, Leonards P, et al. A deep dive into fat: Investigating blubber lipidomic fingerprint of killer whales and humpback whales in northern Norway. *Ecol Evol*. 2021;11(11):6716-6729. doi:10.1002/ece3.7523
4. Jefferson TA, Leatherwood S, Webber MA. Marine Mammals of the world, FAO Species Identification Guide. *FAO and UNEP*. 1993;13(3):587.
5. Yordy JE, Pabst DA, McLellan WA, Wells RS, Rowles TK, Kucklick JR. Tissue-specific distribution and whole-body burden estimates of persistent organic pollutants in the bottlenose dolphin (*Tursiops truncatus*). *Environ Toxicol Chem*. 2010;29(6):1263-1273. doi:10.1002/etc.152
6. North Atlantic Marine Mammals Commission. Killer whale - NAMMCO. Accessed May 20, 2025. https://nammco.no/killer-whale/#1475762140566-81d47f7a-a145
7. Schultes L, van Noordenburg C, Spaan KM, et al. High Concentrations of Unidentified Extractable Organofluorine Observed in Blubber from a Greenland Killer Whale (*Orcinus orca*). *Environ Sci Technol Lett*. 2020;7(12):909-915. doi:10.1021/acs.estlett.0c00661

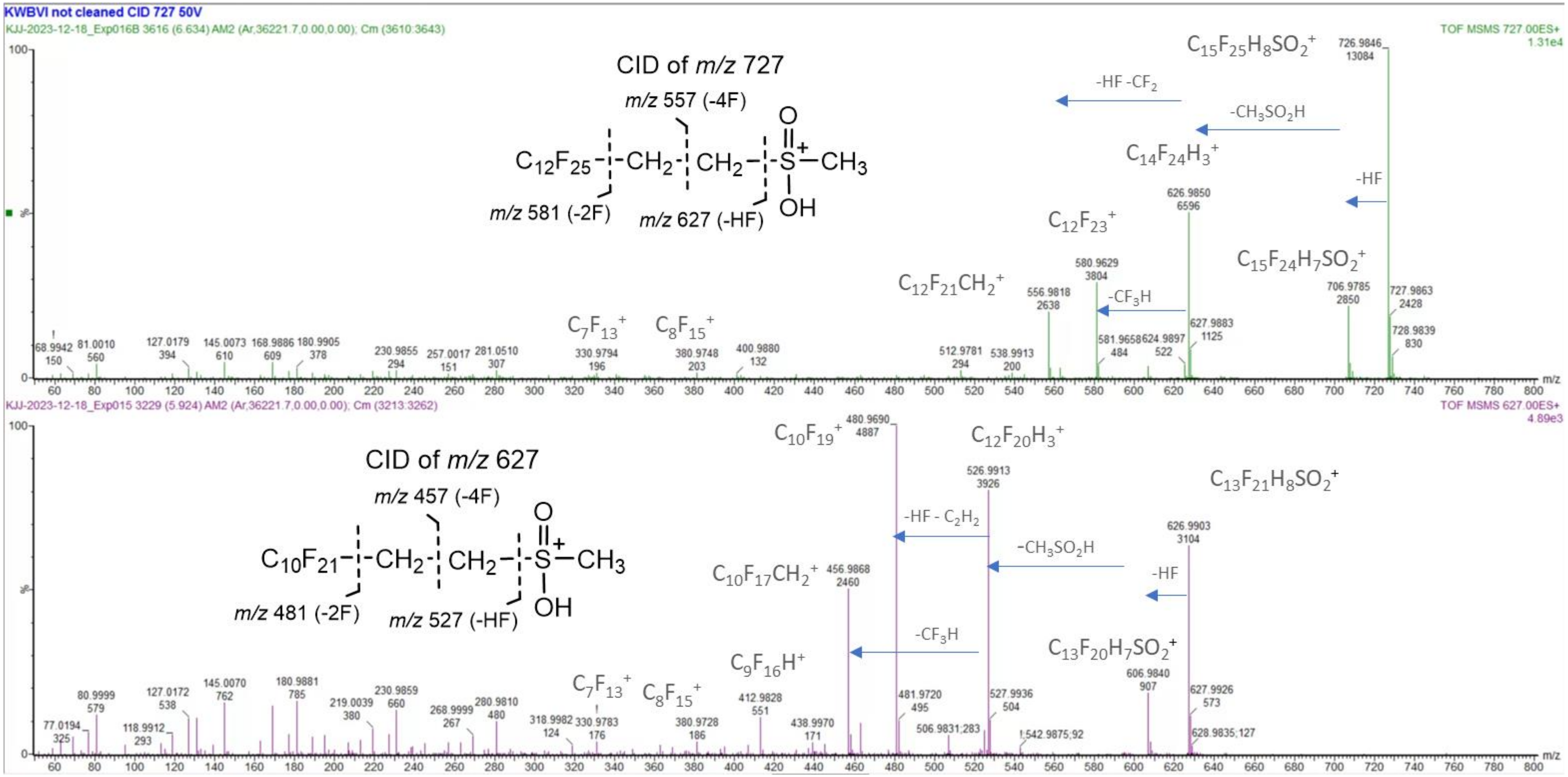

Figure S 1 - MS/MS at 50 V of m/z 727 and 627 in KW-17 and structural assignment as 12:2 and 10:2 fluorotelomer methylsulfones.

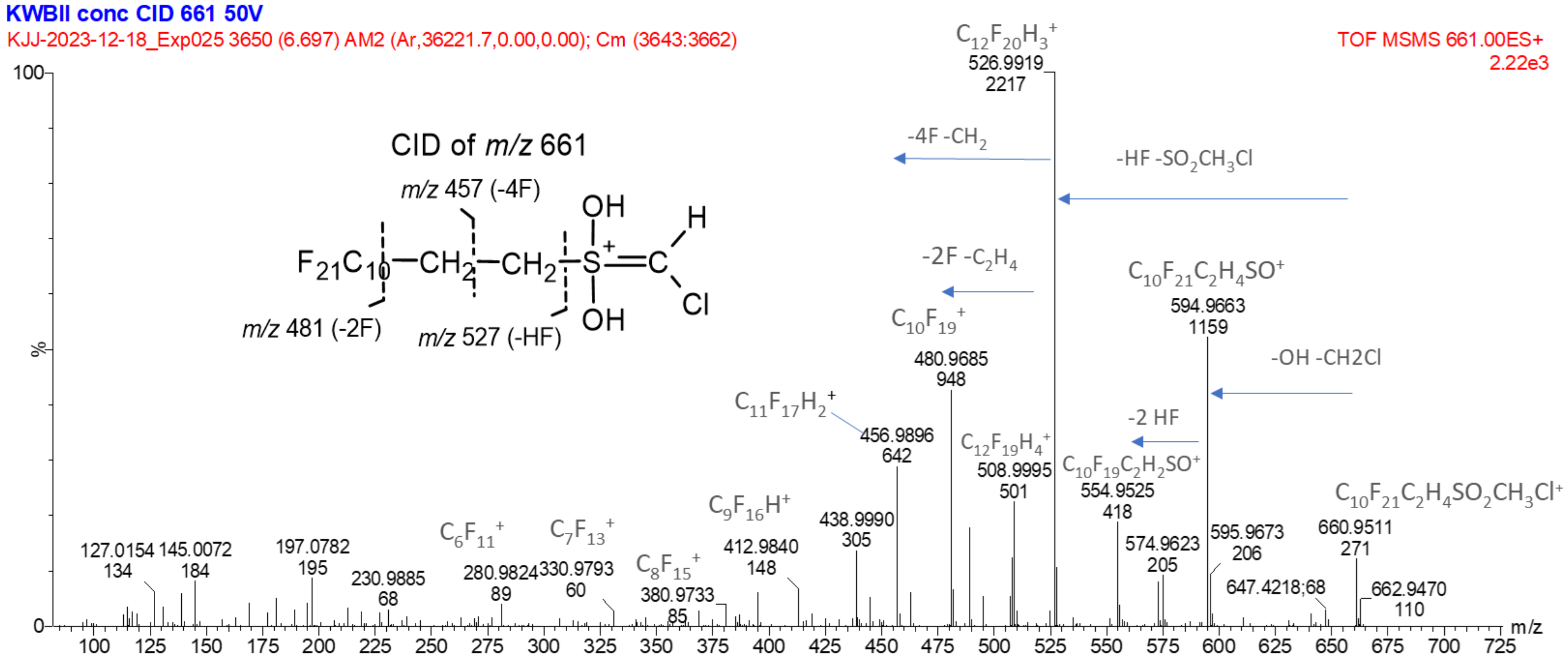

Figure S 2 – MS/MS at 50 V of *m/z* 661 in KW-17 and structural assignment as 10:2 fluorotelomer chloromethylsulfone.

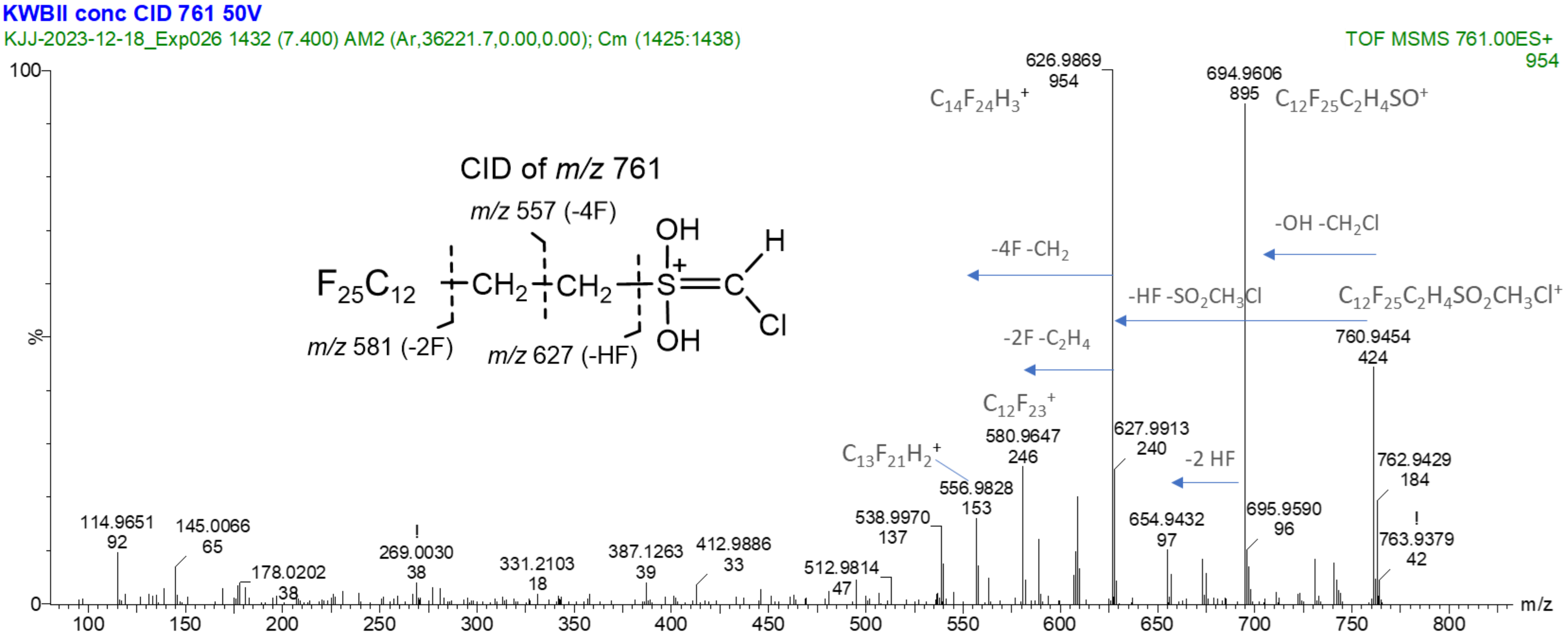

Figure S 3 – MS/MS at 50 V of *m/z* 761 in KW-17 and structural assignment as 12:2 fluorotelomer chloromethylsulfone.

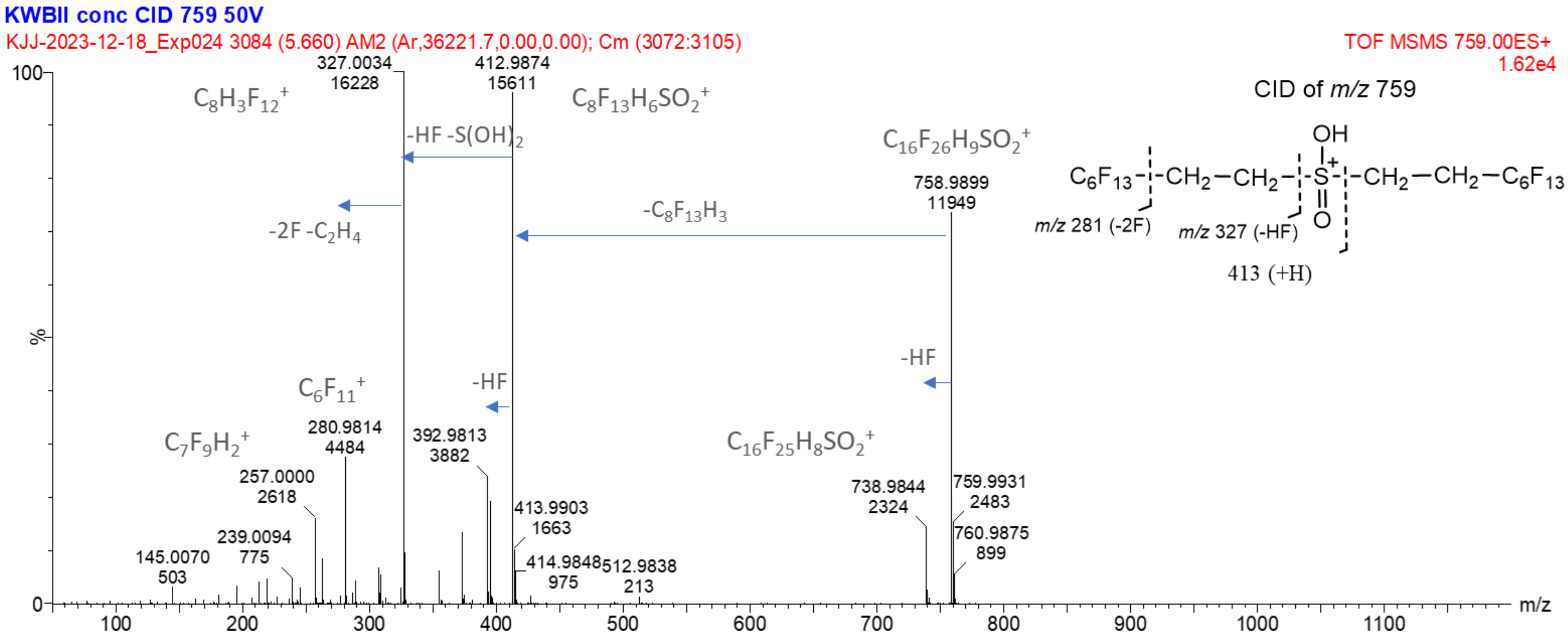

Figure S 4 - MS/MS at 50 V of *m/z* 759 in KW-17 and structural assignment as 6:2 bisfluorotelomer sulfone.

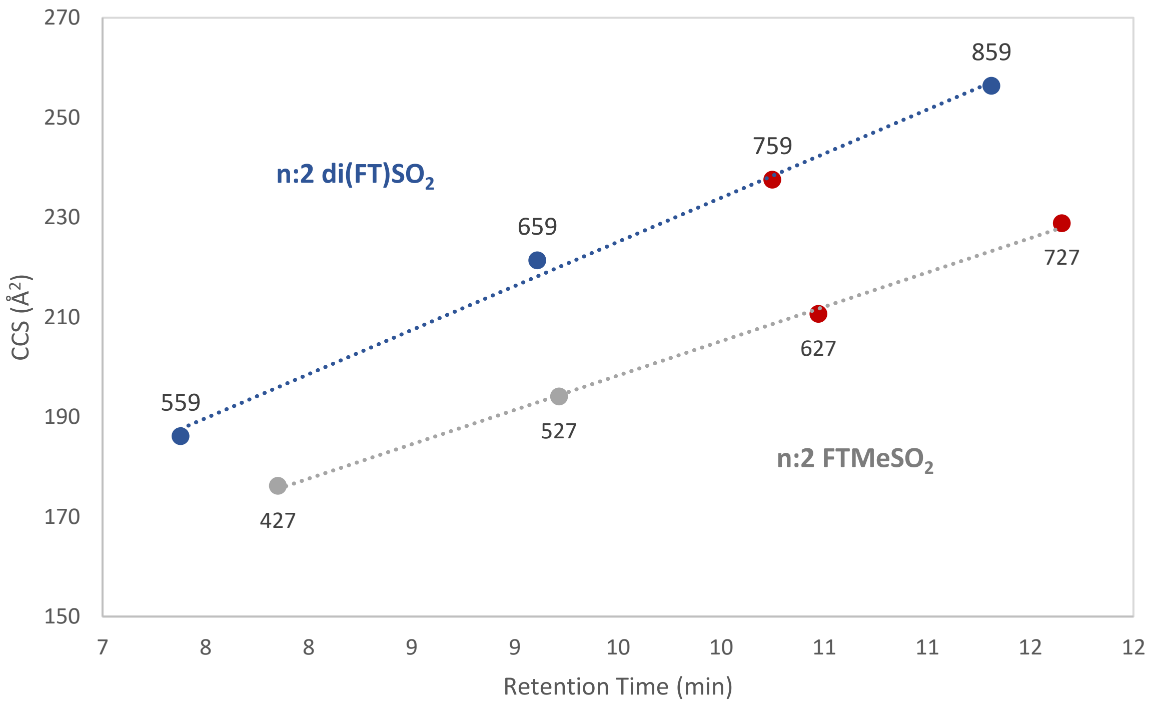

Figure S 5 – Linear trends of retention time versus CCS values for n:2 difluorotelomer sulfone (blue) and n:2 fluorotelomer methylsulfone (grey) homologues. Nominal masses of the homologues are shown next to their respective data points and homologues confirmed with analytical standard are shown in red.

Table S 1 - Information on samples of blubber and liver of killer whales: ID numbers are assigned by Greenland institute of natural resources/Greenland climate research centre or by Swedish museum of natural history, PAX numbers are assigned at Stockholm University. CITES: Convention on International Trade in Endangered Species of Wild Fauna and Flora.

	**IDs**	**Year**	**Sex/Age**	**Sampling Location**	**CITES nr**
KW-16	ID_Oo_001<br>PAX20/0194	18/8/2016	F, subadult	Tasiilaq, Greenland	4.10.18-16603/2020
KW-17	ID_Oo_004<br>ID_2017/0003<br>PAX20/0203 (blubber)<br>PAX20/0205 (liver)	12/09/2017	F, young adult	Tasiilaq, Greenland	17GL1167088
KW-20	ID_C2020/01928<br>PAX20/0103	11/04/2020	F, old adult	Nanortalik, Greenland	20GL1717529
KW-23	ID_A2023/00364<br>PAX24/0001	2023	M, 18 years	Hunnebostrand, Sweden	N/A

Table S 2 – Information on standards of fluorotelomer sulfones purchased from Chiron AS (Trondheim, Norway)

Fluorotelomer sulfone (product name; chemical name; synonyms used in this paper)	Purity	Molecular formula
1H,1H,2H,2H-Perfluoro(methanesulfonyl)dodecane;<br>1,1,1,2,2,3,3,4,4,5,5,6,6,7,7,8,8,9,9,10,10-Henicosafluoro-12-(methylsulfonyl)dodecane;<br>10:2 Fluorotelomer methylsulfone (10:2 $FTSO_2Me$)	99.9±0.11%	$C_{13}H_7F_{21}O_2S$
1H,1H,2H,2H-Perfluoro(methanesulfonyl)tetradecane;<br>1,1,1,2,2,3,3,4,4,5,5,6,6,7,7,8,8,9,9,10,10,11,11,12,12-Pentacosafluoro-14-(methylsulfonyl)tetradecane;<br>12:2 10:2 Fluorotelomer methylsulfone (12:2 $FTSO_2Me$)	96.3±0.11%	$C_{15}H_7F_{25}O_2S$
1H,1H,2H,2H-Perfluoro(chloromethanesulfonyl)dodecane;<br>12-(Chloromethanesulfonyl)-1,1,1,2,2,3,3,4,4,5,5,6,6,7,7,8,8,9,9,10,10-Henicosafluorododecane;<br>10:2 Fluorotelomer chloromethylsulfone (10:2 $FTSO_2MeCl$)	92.7±0.11%	$C_{13}H_6ClF_{21}O_2S$
1H,1H,2H,2H-Perfluoro(chloromethanesulfonyl)tetradecane;<br>14-((Chloromethanesulfonyl)-1,1,1,2,2,3,3,4,4,5,5,6,6,7,7,8,8,9,9,10,10,11,11,12,12-pentacosafluorotetradecane;<br>12:2 Fluorotelomer chloromethylsulfone (12:2 $FTSO_2MeCl$)	96.3±0.11%	$C_{15}H_6ClF_{25}O_2S$
Bis(6:2 perfluorooctyl)sulfone;<br>1,1,1,2,2,3,3,4,4,5,5,6,6-Tridecafluoro-8-((3,3,4,4,5,5,6,6,7,7,8,8,8-tridecafluorooctyl)sulfonyl)octane;<br>6:2 bisfluorotelomer sulfone (bis(6:2 FT)SO_2)	99.9±0.11%	$C_{16}H_8F_{26}O_2S$

Table S 3 - Concentrations in ng/g of the 5 fluorotelomer sulfones, their summed concentrations in ng/g and ng F/g, the EOF concentrations in ng F/g and the % of unknown EOF remaining. Limits of quantification (LOQs) in ng/g for fluorotelomer sulfones and in ng F/g EOF are in the second to last column, while matrix effects are in the last column (expressed as the ratio between the concentration calculated with external one-point calibration and the concentration calculated by standard additions).

**Concentrations**	**KW-17**	**KW-16**	**KW-20**	**KW-23**	**LOQs (ng/g)**	**Matrix Effect (%)**
**10:2 FTSO$_2$Me (ng/g)**	23.63	15.22	15.08	<0.08	0.08	37
**12:2 FTSO$_2$Me (ng/g)**	55.19	38.25	72.46	<0.29	0.29	37
**10:2 FTSO$_2$MeCl (ng/g)**	1.14	0.69	0.92	<0.19	0.19	39
**12:2 FTSO$_2$MeCl (ng/g)**	0.36	<0.28	<0.28	<0.28	0.28	39
**Bis(6:2 FT)SO$_2$ (ng/g)**	3.56	6.17	5.53	<0.13	0.13	62
**$\sum C_{Fluorotelomer\ sulfones}$ (ng/g)**	83.89	60.34	93.99	-	-	-
**$\sum C_{F\_Fluorotelomer\ sulfones}$ (ng F/g)**	54.40	39.16	61.17	-	-	-
**EOF (ng F/g)**	162.3±93.7	69.1	81.6	< 4.7	< 4.7	-
**Unknown EOF (%)**	66.5	43.3	25.0	-	-	-
**$\sum C_{F\_PFAS}$ (ng F/g)***	6.28±0.9	-	-	-	-	-
**Unknown EOF (%)**	62.6		-	-	-	-

Table S 4 – Information on n:2 $FTSO_2Me$ and n:2 $diFTSO_2$ homologue series. Homologues confirmed with analytical standards are in bold. The consistent increasing retention time (RT) and collision cross section (CCS) values with the increase of m/z give additional evidence of a homologue series. Abundances reported here were obtained from Progenesis QI, peaks were manually inspected in MassLynx and only peaks with good shape and signal to noise above 10 (S/N > 10) are reported.

	Homologue	theoretical m/z	measured m/z	ppm error	RT (min)	CCS (Å^2)	KW-16	KW-17	KW-20	KW-23
n:2 $FTSO_2Me$	6:2	427.0032	427.0042	2.3	7.85	176.1	6	23	5	
	8:2	526.9968	526.9985	3.1	9.21	194.0	59	262	24	14
	**10:2**	**626.9904**	626.9943	1.1	10.47	210.6	1234	10683	1427	
	**12:2**	**726.9840**	726.9881	0.9	11.65	228.8	1081	7517	1749	
n:2 $diFTSO_2$	4:2	559.0030	558.9966	-11.6	7.38	186.1	14		6	
	5:2	658.9967	658.9985	2.8	9.11	221.3	6	53	5	
	**6:2**	**758.9903**	758.9931	1.4	10.25	237.5	407	1000	331	
	7:2	858.9839	858.9861	2.6	11.31	256.3	156	134	105	
